# Mental Nerve Anterior Loop Detection in Panoramic and Cone Beam Computed Tomography Radiograph for Safe Dental Implant Placement

**DOI:** 10.7759/cureus.30687

**Published:** 2022-10-25

**Authors:** Badr Othman, Talal Zahid

**Affiliations:** 1 Periodontology Department, Faculty of Dentistry, King Abdulaziz University, Jeddah, SAU

**Keywords:** digital panoramic radiographs, mandibular incisive nerve, diagnose, factors determining dental implant therapy, accessory mental foramen (amf)

## Abstract

Background and aim

Different imaging modalities have been used as preoperative assessment tools since the emergence of dental implants. This study aimed to compare the detection and presence of mental nerve anterior loop in cone beam computed tomography (CBCT) radiograph and panoramic radiograph.

Material and methods

A descriptive, retrospective study was done. The optimal sample size was calculated using Epi Info software. According to the study population (795), the sample size was 259 cases which gave 95% power of the study. A previously taken CBCT and digital panoramic radiographs from the database of Taibah University Dental School and Hospital were observed by two trained and calibrated examiners to determine the presence of a mental nerve anterior loop and compare the two modalities. The mean length of the mental nerve anterior loop was also assessed.

Results

Mental nerve anterior loops were detected bilaterally in 57.1% and 17.4% using CBCT and panoramic radiographs, respectively. In CBCT, the right side (20.8%) had a higher prevalence of mental nerve anterior loop than the left side (12%). The mental nerve anterior loop was not visible in 49.4% of the cases using a panoramic radiograph, while CBCT gave 10% of cases as not visible. The mental nerve anterior loop average length was 1.8 ± 0.35 mm and the width was 1.7 ± 0.28 mm. The average distance from the mental foramen to the inferior border of the mandible was 12.1 ± 0.87 mm and the average distance from the mental frogmen to the mandibular midline was 25.1 ± 0.68 mm.

Conclusion

As differences between CBCT and panoramic radiographs were statistically significant, CBCT is more accurate and reliable. CBCT is recommended to be used as a preoperative assessment tool to minimize nerve injury-related surgical complications during implant placement at the mandibular premolar area.

## Introduction

In the 1980s, a new treatment modality has been introduced to the field of dentistry, called a dental implant. High success rates (94-100) have been associated with these implants, which makes it the treatment of choice for replacing missing teeth [1]. As with any other prosthetic device placed in the human body, dental implant placement can be challenging. Good diagnosis and treatment planning is mandatory to achieve success.

One of the most important considerations during treatment planning is adjacent vital structure. In the maxilla, the nasal floor and maxillary sinus are the main landmarks responsible for postoperative complications [2]. Mandibular landmarks include mandibular foramen, inferior alveolar nerve, mental and lingual nerves [3]. Surgical procedures must avoid any disturbance to these structures.

The mental nerve is an anterior continuation of the inferior alveolar nerve. Before the mental nerve passes out through the mental foramen, it may form an anterior loop. Anterior loop of the mental nerve is defined in Sicher's Oral Anatomy as “the mental canal which rises from the mandibular canal and runs outward, upward, and backward to open at the mental foramen.” this specific structure may limit Implant placement in the interforaminal area. Neurosensory disturbance to the chin and lower lip arises as a result of trauma to the mental nerve from implant placement [4].

There are several imaging modalities that can be used to detect and measure the anterior loop. These are panoramic radiography, cone beam computed tomography (CBCT), spiral CT, and MRI [5]. Commonly, panoramic radiography and CBCT are used for preoperative assessment by clinicians.

Panoramic radiography is one of the major imaging systems in dental radiology, in which radiographs show a full image of jaws and maxillofacial structures [6]. Panoramic radiograph has different applications in dentistry such as periodontal evaluation, orthodontic treatment planning, oral surgery, and implant treatment planning [7]. As a 2D image, a panoramic radiograph has several shortcomings like magnification, distortion, superimposition, and misrepresentation of structures and can miss displaced mandibular fractures as well [8]. In contrast, CBCT is a 3D imaging system. Rather than a single image, CBCT creates sub-millimeter spatial resolution images. Within a short scanning time (10-70 seconds), clinicians can get a high diagnostic quality image, which is helpful to accurately visualize the area of interest. Comparing CBCT radiation dose to panoramic radiograph, one CBCT is equivalent to 4-15 panoramic radiographs [9].

In implant treatment planning in the mental region, some clinicians claim it is relatively safe, and a panoramic radiograph can be used for that purpose since the magnification is small in the anterior region [10]. However, for others, distortion and magnification of anatomical structures are often present in the panoramic radiograph even in the anterior region. Based on that, the results are either over or underestimation of the real size [4].

In Vujanovic-Eskenazi's study, they recommended CBCT to be used when planning for implant in the anterior region as a 2D panoramic radiograph imaging was less accurate and reliable due to its magnification. However, in their results, CBCT and panoramic radiograph difference was not statistically significant [4]. In another study done by Couto-Filho et al., the anterior loop was detected in 42.6% and 29.8% of cases in panoramic radiograph and CBCT, respectively. According to the statistical comparison, a panoramic radiograph led to a false positive diagnosis of the anterior loop [11]. It is not reasonable to have a general safe margin for implant placement in the anterior region due to individual variations as mentioned by Brito et al. [12]. While Vujanovic-Eskenazi et al. recommended a larger sample size to be able to identify a safety margin [4]. CBCT showed higher sensitivity and specificity for implant complications when compared to panoramic radiographs [13]. On the other hand, no significant difference was found in the visualization of the anterior loop between the panoramic radiograph and CBCT [12].

The dental clinics equipment may vary depending on its size, location, and demand. Medina city is the main city in Saudi Arabia with a large population. However, most dental clinics are less equipped with general dentists dominating the dental market when compared to other dental clinics in the main cities. The study objective is to tackle the importance of the presence of CBCT machines as a standard of care in dental clinics that practice dental implants to avoid complications. The anterior loop was specifically chosen as a comparison due to its unclear location, extension, and visibility, increasing the chance of hitting the loop with iatrogenic dentistry.

The aim of this study is to detect and assess the visibility of the anterior loop on both panoramic radiographs and CBCT. In order to determine which is more applicable to be used as a pre-operative assessment tool for implant placement in the premolar region without inducing complications related to the anterior loop of the mental nerve.

## Materials and methods

Study design and setting

Ethical Approval was obtained from Taibah University, College of Dentistry Research Ethics Committee (TUCDREC). A descriptive, retrospective study was done for the patient database at the radiology unit of Taibah University Dental College and Hospital, Madinah, Saudi Arabia was conducted. The study was performed over a period of six months.

Sample size, inclusion criteria, and exclusion criteria

The optimal sample size was calculated using Epi Info software. The total number of cases found in the database was 795. According to the study population (795), the sample size was 259 cases which gave 95% power of the study. Two examiners were trained and calibrated to look for all CBCT available in the radiology unit. Intra-examiner and inter-examiner reliability test was done through a pilot of 10 cases after a demonstration case. The results were consistent within the same examiner and between both of them. Inclusion criteria were both male and female patients ranging from 20 to 70 years old who had both CBCT and OPG taken previously for different reasons. Exclusion criteria were patients with the implant in the mandible “which produce an artifact in the image,” fracture in the mandible, local or systemic osseous disease in the anterior mandible and sectional CBCT, any patient with a history of trauma to the anterior part of the mandible and any history of surgical procedure or cystic lesion in the premolar area. CBCT and OPG images were obtained from the same machine (Kodak 9000 3D, Carestream Health, Inc., NY, USA), and only for patients were exposed to both images.

Measurements and radiographic assessment 

**Figure 1:**
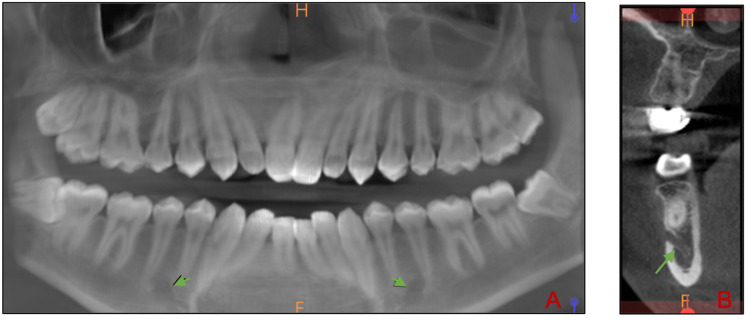
Detection of the anterior loop using CBCT (A) Anterior loop detected bilaterally (green arrows) in the reconstructive panoramic view from CBCT. (B) The cross-sectional slice shows an anterior loop.

**Figure 2:**
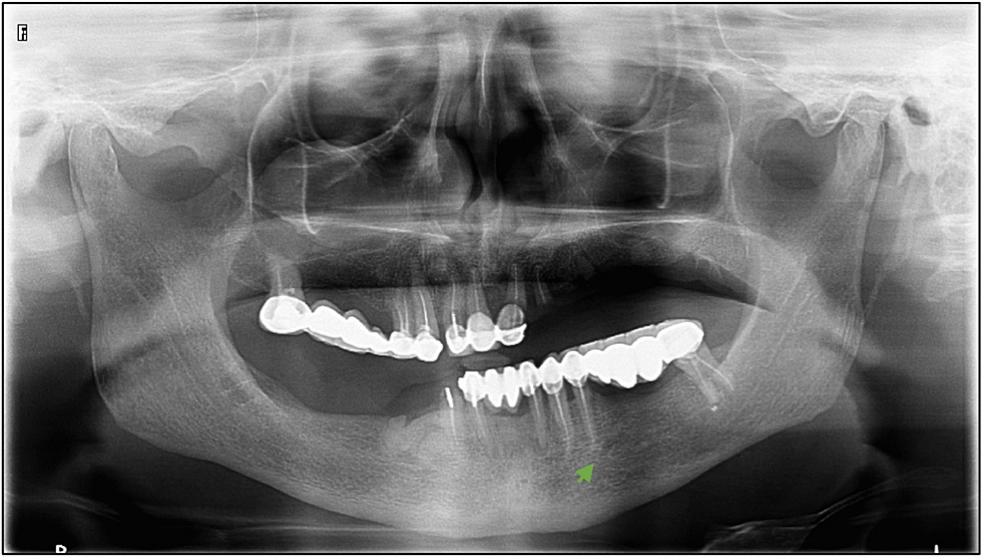
Anterior loop detection in a panoramic radiograph, anterior loop detected on left side (green arrow)

Using Carestream Dental CS imaging 7 software, the examiners assessed both CBCT and panoramic radiographs in terms of the presence and detection of the anterior loop of the mental nerve. The anterior loop is identified as the visible bilateral, unilateral right side, unilateral left side, or not visible (Figures 1A, 1B, 2). The nerve tracing feature was used during the CBCT analysis. Panoramic and cross-sectional planes with 1 mm thickness were used to detect the anterior loop.

 

**Figure 3:**
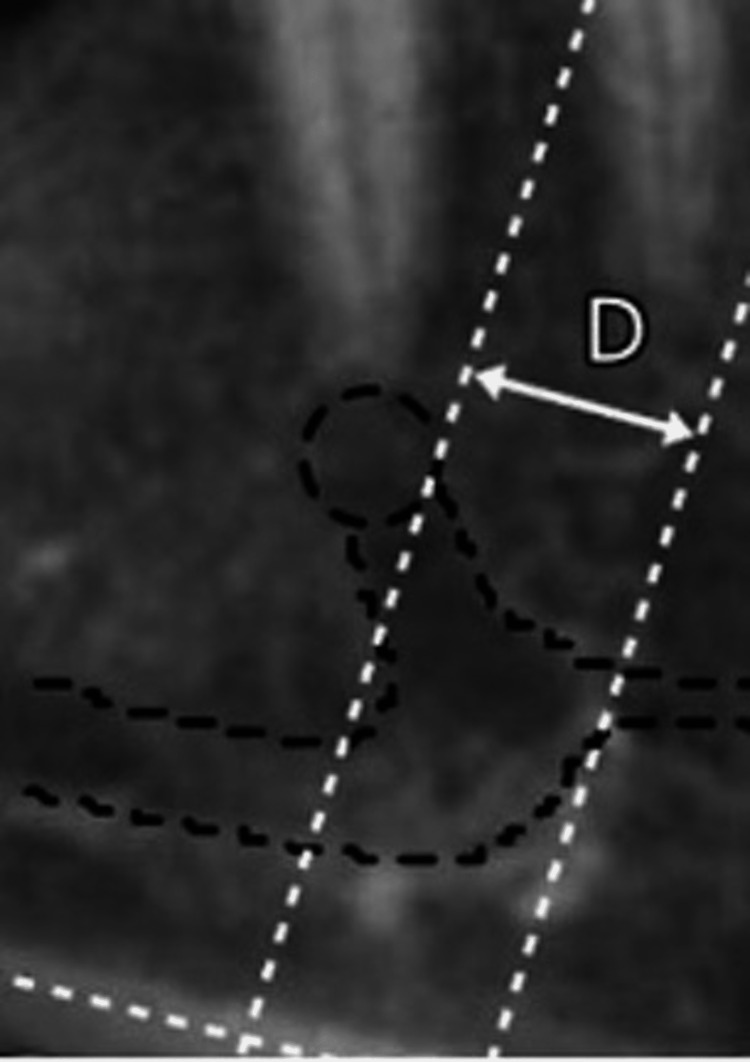
Anterior loop anterior average length

**Figure 4:**
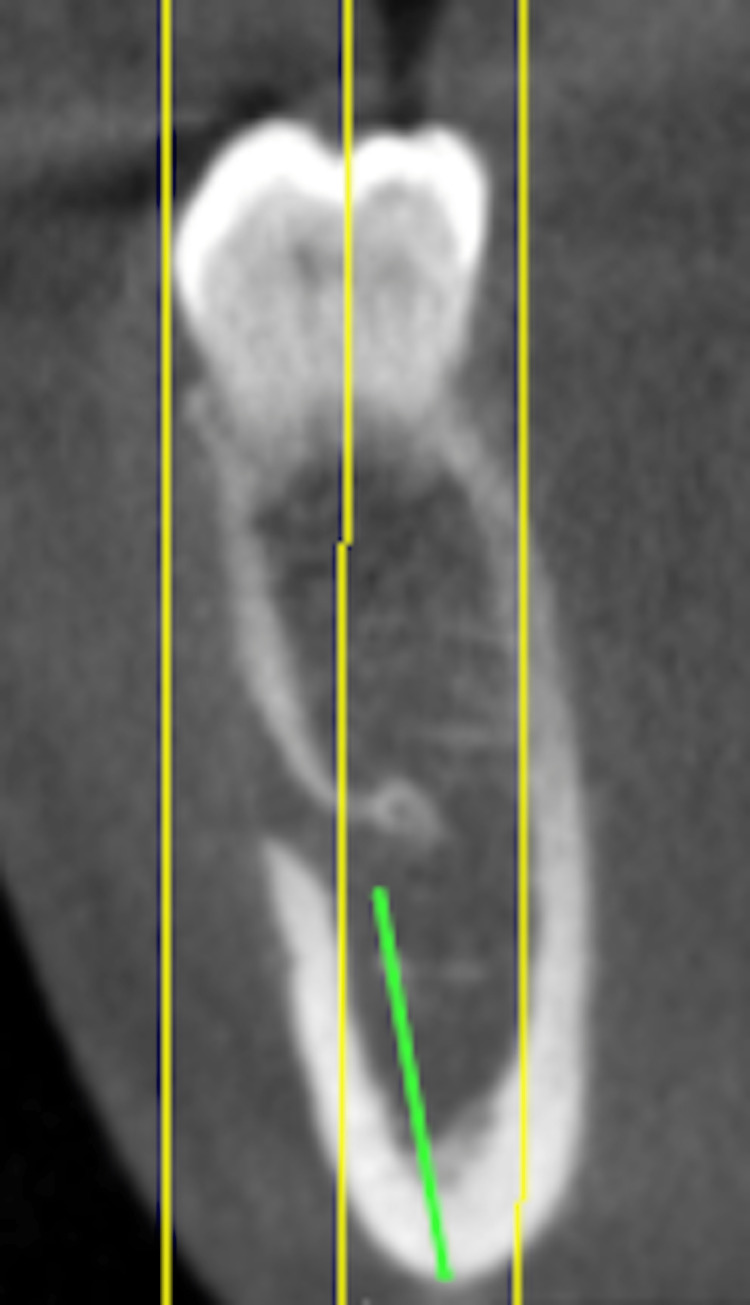
The average distance from the mental nerve to the inferior border of the mandible

Additionally, the anterior loop average length, mental foramen average width and the average distance from the mental nerve to the inferior border of the mandible, and the average distance from the mental nerve to the mandibular midline were measured (Figures 3, 4).

## Results

Demographics

**Figure 5:**
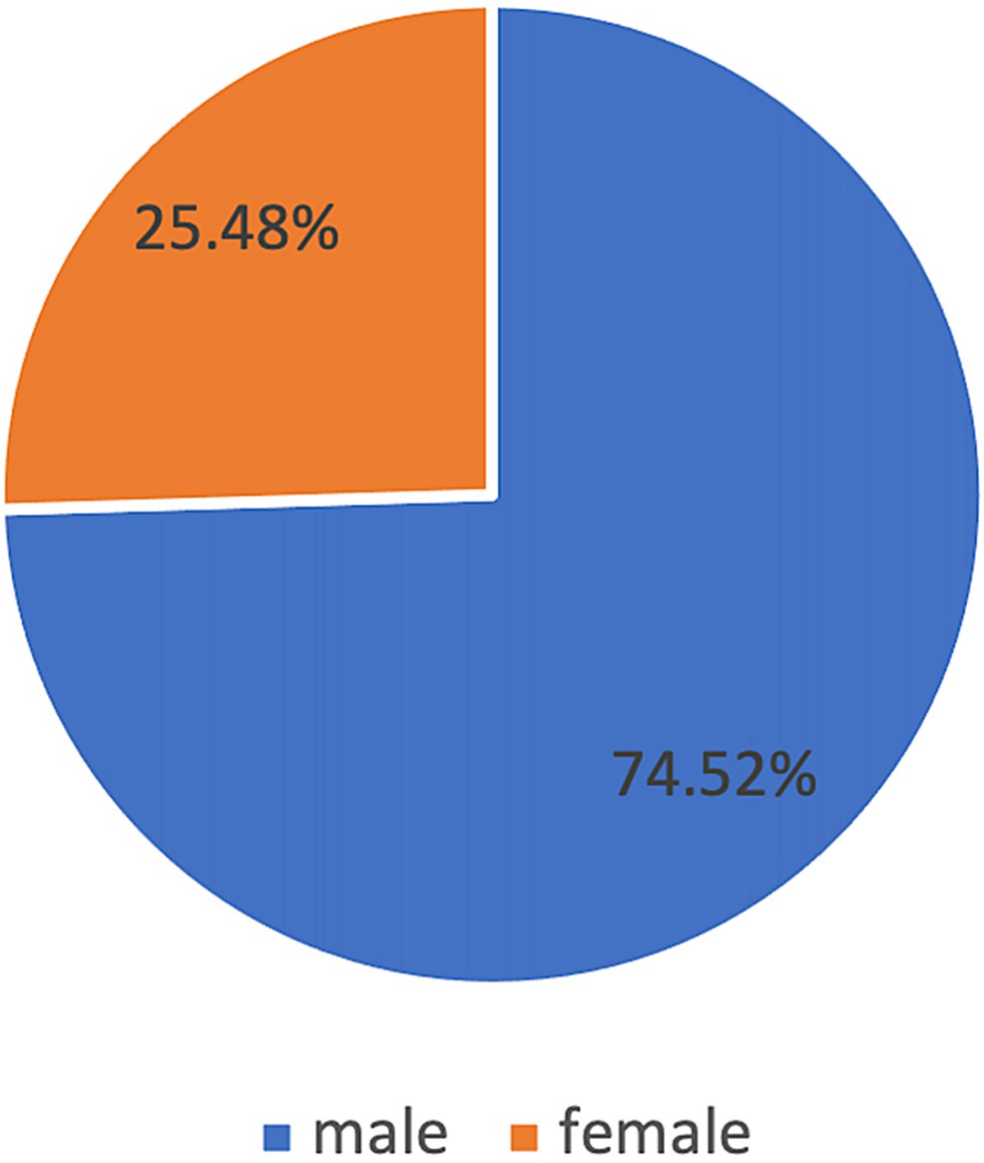
Percentage of male and female patients among the radiographic study sample

A total of 259 patients were included in this study. The percentage of male and female patients was 75% and 25%, respectively (Figure 5). There was no significant difference between the right and left sides or between gender in all measurements. The mean age was 46 ± 13.

Anterior loop in CBCT 

As the visibility of the anterior loop was assessed in both sides, in most of the cases (57%) anterior loop was detected bilaterally in CBCT and only few cases (10%) showed complete absence of the loop. the visibility of the anterior loop on the right side was significantly higher than on the left side (P = 0.002; Table 1). Moreover, differences between genders were not statistically significant.

**Table 1:** Anterior loop visibility in CBCT *P=0.002, Pearson Chi-square

Anterior loop visibility in CBCT	Number of cases, n (%)
Visible bilaterally	148 (57.1%)
Visible unilaterally right side*	54 (20.8%)
Visible unilaterally left side*	31 (12%)
Not visible/ absent	26 (10%)

Anterior loop in panoramic radiograph

The visibility of the anterior loop bilaterally was only 17.4% and it could not be detected in 49.4% of the cases. Anterior loop visibility on the right side was significantly higher than on the left side (P = 0.002; Table 2).

**Table 2:** Anterior loop visibility in panoramic radiograph P=0.002, Pearson Chi-square

Anterior loop visibility in panoramic radiograph	Number of cases, n (%)
Visible bilaterally	45 (17.4%)
Visible unilaterally right side*	50 (19.3%)
Visible unilaterally left side*	36 (13.9%)
Not visible/ absent	128 (49.4%)

Comparison between CBCT and panoramic radiograph

In this study, two observers were trained to detect anterior loop of the mental nerve in CBCT and panoramic radiograph. Kappa statistics showed 0.895 (P = 0.001) and 0.726 (P = 0.001) reproducibility for CBCT and panoramic radiograph, respectively. The chi-squared test found the visibility of the anterior loop bilaterally in CBCT is significantly higher than in panoramic radiographs. There was no significant difference between CBCT and panoramic radiographs in cases where the anterior loop was detected only unilaterally on the right or the left side. Anterior loop absence was significantly higher in panoramic radiographs when compared to CBCT.

Radiographic assessments

Based on the CBCT image assessment, the anterior loop average length was 1.8 ± 0.35 mm and the mental foramen average width was 1.7 ± 0.28 mm. The average distance from the mental foramen to the inferior border of the mandible was 12.1 ± 0.87 mm and the average distance from the mental foramen to the mandibular midline was 25.1 ± 0.68 mm.

## Discussion

A preoperative radiograph is mandatory for dental implant treatment planning. It aids in the determination of location, number, angulation, and appropriate size for implant. It is even more important for the safety of vital structures. To our best knowledge, a comparison between panoramic radiographs and CBCT in the detection and measurement of the anterior loop has been discussed in a limited number of studies. The anterior loop of the mandibular canal is a source of possible complications due to the mesial extension of the inferior alveolar nerve [14].

In the present study, the anterior loop was detected bilaterally in 57.1% of the cases in CBCT, while the panoramic radiograph showed an anterior loop bilaterally in 17.4% of the cases. Similar to De Brito et al.'s study and Vujanovic-Eskenazi et al.'s study, the difference in the detection of the anterior loop between panoramic radiograph and CBCT was statistically significant [4,12].

Our results were in agreement with Vujanovic-Eskenazi et al.'s study (48.8%) and Apostolakis and Browns’ study (48%), as a higher detection rate was found using CBCT comparing it to panoramic radiograph [4,15]. Although, opposite results were found by of Couto-Filho et al. and De Brito et al., which can be explained due to a smaller sample size than current and previous supportive studies and they justified their result as there could be low visibility of the anterior loop in their population in general [11,12]. Differences in detection rate between studies are shown in Table 3. These findings are considered a guiding sign that using CBCT for implant placement in the anterior region is more reliable and precise than a panoramic radiograph.

**Table 3:** Anterior loop detection rates in different studies

Study	Year	Location	Sample size	CBCT	Panoramic radiograph
Vujanovic-Eskenazi et al. [4],	2015	School of Dentistry, University of Barcelona, Barcelona, Spain. Researcher of the IDIBELL Institute Barcelona, Spain	82	48%	36.6%
De Borito et al. [11]	2016	Piracicaba Dental School, University of Campinas, São Paulo, Brazil.	90	7.7%	15%
Couto-Filho et al. [10]	2015	Piracicaba Dental School, University of Campinas, São Paulo, Brazil.	94	29.8%	42.6%
Present study	2022	Taibah University Dental School and Hospital, Medina, Saudi Arabia	259	57.1%	17.4%

Using CBCT to detect the anterior loop seemed to be easier for our observers as they could visualize the loop in different sections and views. However, panoramic reconstruction from CBCT was clearer than the cross-sectional view, the same result was noticed by Kaya et al. [16]. Vujanovic-Eskenazi et al. disagreed as the loop was identified using a parasagittal view [4]. This diversity brings out one of CBCT's advantages, as a variety of views make it more convenient for different clinicians to identify the most reliable and accurate one to be used for assessment.

Although Mardinger et al.’s study estimated an anterior loop detection rate of as much as 40% using panoramic radiographs, dissection of radiographed anatomical specimens proved false positive results [17]. This finding coincides with Kuzmanovic et al., as they concluded that identifying the anterior loop using a panoramic radiograph was associated with a high incidence of false positive and false negative results which renders a panoramic radiograph an unreliable assessment tool [18]. Several years later, Couto-Filho et al. followed with the same approach and concluded with a similar finding [11].

The anterior loop was not visible in almost half of our sample using a panoramic radiograph (49.4%). However, in Vujanovic-Eskenazi et al.'s sample (63.4%), the anterior loop was not visible [4]. Despite percentages, even a minor number of cases with the possibility of false negative results using panoramic radiographs eliminate its use as a reliable preoperative assessment tool.

In line with this study, De Brito et al.'s study and Lu et al.’s study statistical difference was not identified based on gender [12,19]. Regarding this study, CBCT yielded a higher detection rate of the anterior loop on the right side (20.8%) than on the left side (12%). This was similar to Couto-Filho et al.'s study and De Brito et al.’s study [11,12]. In contrast, Lu et al.’s study revealed no statistically significant difference between both sides [11].

Moreover, the Kappa value was calculated for reproducibility. In our study, CBCT showed a higher Kappa value than the panoramic radiograph. These results were similar to Vujanovic-Eskenazi et al.'s study [4]. This indicates that CBCT is more reliable to be used as a preoperative assessment tool.

In general, the present study showed a high frequency of the anterior loop, in contrast to Couto-Filho et al.'s study, which identified a low frequency of the anterior loop [11]. Although this study has included a larger sample size compared to previous studies, one of the limitations is that the age factor was not included in the variables since it was not available in the radiology unit.

As Vujanovic-Eskenazi et al.'s study recommended a larger study sample size to determine the general safety margin [4]. De Brito et al.'s study showed that there is no general safe zone for implant placement in the anterior region [12]. This study agreed with the latter results.

One of the panoramic radiographs advantages is to give an overview of the dentition and jaws. It has limited advantages to assess implant surgeries due to its 2D view and the distortions in the horizontal plane and magnification of the vertical plane [20]. On the other hand, CBCT can give highly accurate images of the patient's anatomy. A well-detailed image is a tool for good diagnosis and management to minimize complications [21].

In our study, we investigated the presence of the anterior loop as it is one of the challenging anatomical features of the inferior alveolar nerve. This may increase the complications of the dental implant placement in the premolar region of the mandible. Panoramic radiographs solely are not advised to assess, and plan implants due to their limitations. Dental implant practice clinics should be equipped with CBCT as it became the new standard of care.

## Conclusions

In the literature, anterior loop detection showed discrepancies between CBCT and panoramic radiographs. This discrepancy led a variety of studies together with our study to recommend the use of CBCT as a safe preoperative assessment tool before implant placement in the mandibular premolar region. A panoramic radiograph can provide limited benefit in the detection of the anterior loop of the mental nerve, which may increase the incidence of anterior loop violation. Further studies are needed for the measurements related to the anterior loop such as distance from the inferior border of the mandible and length of mesial extension using CBCT to determine averages in different races rather than safe zone.

## References

[1] Rokn AR, Hashemi K, Akbari S, Kharazifard MJ, Barikani H, Panjnoosh M (2016). Accuracy of linear measurements using cone beam computed tomography in comparison with clinical measurements. J Dent (Tehran).

[2] Prasad K, Shetty M, Mehra D (2013). Anatomical consideration in implant selection and positioning. Int J Oral Implantol Clin Res.

[3] Cooper S (2015). The anatomical landmarks most important for dental implant surgery. J Human Anat Physiol Soc.

[4] Vujanovic-Eskenazi A, Valero-James JM, Sánchez-Garcés MA, Gay-Escoda C (2014). A retrospective radiographic evaluation of the anterior loop of the mental nerve: comparison between panoramic radiography and cone beam computerized tomography. Med Oral Patol Oral Cir Bucal.

[5] Neves FS, Nascimento MC, Oliveira ML, Almeida SM, Bóscolo FN (2014). Comparative analysis of mandibular anatomical variations between panoramic radiography and cone beam computed tomography. Oral Maxillofac Surg.

[6] Scarfe WC, Farman AG (2008). What is cone-beam CT and how does it work?. Dent Clin North Am.

[7] Pittayapat P, Galiti D, Huang Y (2013). An in vitro comparison of subjective image quality of panoramic views acquired via 2D or 3D imaging. Clin Oral Investig.

[8] Albassal A, Al-Khanati NM, Harfouch M (2021). Could a digital panoramic x-ray not detect a displaced fracture of the mandible?. Quant Imaging Med Surg.

[9] Scarfe WC, Farman AG, Sukovic P (2006). Clinical applications of cone-beam computed tomography in dental practice. J Can Dent Assoc.

[10] Raitz R, Shimura E, Chilvarquer I, Fenyo-Pereira M (2014). Assessment of the mandibular incisive canal by panoramic radiograph and cone-beam computed tomography. Int J Dent.

[11] do Couto-Filho CE, de Moraes PH, Alonso MB, Haiter-Neto F, Olate S, de Albergaria-Barbosa JR (2015). Accuracy in the diagnosis of the mental nerve loop. A comparative study between panoramic radiography and cone beam computed tomography. Int J Morphol.

[12] de Brito AC, Nejaim Y, de Freitas DQ, de Oliveira Santos C (2016). Panoramic radiographs underestimate extensions of the anterior loop and mandibular incisive canal. Imaging Sci Dent.

[13] Guerrero ME, Noriega J, Jacobs R (2014). Preoperative implant planning considering alveolar bone grafting needs and complication prediction using panoramic versus CBCT images. Imaging Sci Dent.

[14] Gómez-Román G, Lautner NV, Goldammer C, McCoy M (2015). Anterior loop of the mandibular canal-a source of possible complications. Implant Dent.

[15] Apostolakis D, Brown JE (2012). The anterior loop of the inferior alveolar nerve: prevalence, measurement of its length and a recommendation for interforaminal implant installation based on cone beam CT imaging. Clin Oral Implants Res.

[16] Kaya Y, Sencimen M, Sahin S, Okcu KM, Dogan N, Bahcecitapar M (2008). Retrospective radiographic evaluation of the anterior loop of the mental nerve: comparison between panoramic radiography and spiral computerized tomography. Int J Oral Maxillofac Implants.

[17] Mardinger O, Chaushu G, Arensburg B, Taicher S, Kaffe I (2000). Anterior loop of the mental canal: an anatomical-radiologic study. Implant Dent.

[18] Kuzmanovic DV, Payne AG, Kieser JA, Dias GJ (2003). Anterior loop of the mental nerve: a morphological and radiographic study. Clin Oral Implants Res.

[19] Lu CI, Won J, Al-Ardah A, Santana R, Rice D, Lozada J (2015). Assessment of the anterior loop of the mental nerve using cone beam computerized tomography scan. J Oral Implantol.

[20] Ramakrishnan P, Shafi FM, Subhash A, Kumara A, Chakkarayan J, Vengalath J (2014). A survey on radiographic prescription practices in dental implant assessment among dentists in Kerala, India. Oral Health Dent Manag.

[21] Nehaanilpatil Nehaanilpatil, Gadda R, Salvi R (2012). Cone beam computed tomography: adding the third dimensions. J Contempor Dentistry.

